# Caring for children with spinal muscular atrophy in Greece: parents' and caregivers' experience with the healthcare system

**DOI:** 10.3389/frhs.2025.1612270

**Published:** 2025-10-23

**Authors:** Kyriakos Souliotis, Christina Golna, Chara Tzavara, Christiana Vasileiadi, Pavlos Golnas, Aikaterini Ntokou, Kornilia Binou, Antigone Karras

**Affiliations:** ^1^ Department of Social & Educational Policy, University of Peloponnese, Corinth, Greece; ^2^ Health Policy Institute, Maroussi, Athens, Greece; ^3^ MDA Hellas, Athens, Greece

**Keywords:** patient journey, spinal muscular atrophy, disease burden, quality of life, health policy, patient advocacy

## Abstract

**Introduction:**

Spinal muscular atrophy (SMA) is a rare disease characterized by challenges in its management both for patients and parents/caregivers. In Greece, these challenges are exacerbated by the lack of an integrated SMA patient pathway. This survey mapped the experiences of parents of SMA pediatric patients with the Greek healthcare system, highlighting the actual pathway, its impact on quality of life, and associated financial burden.

**Methods:**

Parents of children living with SMA, who were members of MDA Hellas, a non-profit organization for people with neuromuscular diseases in Greece, were invited to complete a novel online questionnaire, prepared in collaboration with the Health Policy Institute, a non-profit research organization specializing in health policy evaluation and design. The survey ran from May to September 2023. The results were analyzed using SPSS V26.0.

**Results:**

Forty-four parents participated in the survey, with a mean age of 43.2 years. In the absence of universal prenatal or newborn screening, parents were the first to recognize any symptoms in the majority (73.5%) of cases. A total of 38.6% of parents had to travel from their place of residence to receive a diagnosis for their child. More than half had to pay out-of-pocket for diagnosis-related services. Parents shouldered additional treatment services and assistive aids' costs in excess of 300 euros a month. The majority reported feelings of agony and fear (90.9%) and guilt (25%) upon receiving the diagnosis. The average PedsQol score was 67 (SD = 15.8), whereas Zarit scores confirmed a higher burden for caregivers of children with graver SMA types.

**Conclusion:**

This survey details the concerns and needs and quantifies SMA burden on parents/caregivers of children with SMA in Greece to inform health policy advocacy. Such advocacy should be translated into a national action plan with a focus on integrating care organization and delivery options.

## 1 Introduction

Spinal muscular atrophy (SMA) is a severe neurodegenerative condition caused by recessive mutations in the survival motor neuron (SMN) 1 gene, resulting in insufficient SMN proteins. It is considered a rare disease, with a prevalence of 1–2 cases per 100,000 persons and an incidence of 1 in 10,000 live births (1). SMA leads to progressive muscle weakness, atrophy, and multisystem physical dysfunction, which severely impacts patients' quality of life (2). Recent, rapid advances in diagnosis and treatment have significantly changed the clinical picture of the disease (3), yet timely access is of paramount importance for patients to reap any benefits.

The disease's management pathway across the public health continuum should include the following areas: (a) prevention and early diagnosis, including preconception, prenatal and newborn screening; (b) in the absence of universal screening, symptoms' recognition and referral to diagnostic services; (c) diagnosis and linkage to care; (d) care delivery, including a wide array of health and welfare services and education; and (e) transition to adult care. Across these areas, patients and their caregivers journey through (i) services, which include access to health, welfare, educational, and other supportive services; (ii) feelings and needs, and what would have probably sustained or helped them; and (iii) costs, which they might have to incur to ensure access to and delivery of services.

This journey is further aggravated by the nature of SMA as a rare disease. In rare diseases, where the population is small and geographically dispersed, harmonizing management processes and practices is difficult, as the health system lacks the critical demand mass to both prioritize and streamline its function to address patient needs. This compounds the challenges faced by patients with SMA and their parents and caregivers, adding to their burden.

To ameliorate some of these challenges and provide a universally agreed minimum set of processes and outcomes that should be targeted during each area of the disease management continuum, standards of care were agreed upon among the global scientific community (4–6). These are supplemented by emerging literature (7) guiding ongoing management of SMA, as various therapies and processes become available, and the disease paradigm evolves to incorporate them.

Nonetheless, in Greece, such standards are hard to uphold. Healthcare services struggle with integration (8), with the geography of the country further undermining equity in access to care (9), and staff shortages, whereas patients are faced with high out-of-pocket payments (10), fueled by years of healthcare system underfunding (11). The situation was further exacerbated by the economic crisis and the COVID-19 pandemic, which have both, in sequence, tested the sustainability of the system. For the 150 people, children and adults, living with SMA in Greece and registered with MDA Hellas, and the additional 1–6 live births (8 per 100,000 live births) per annum (12), the struggle with overcoming these barriers may be even harder. This is due to the fact that, despite the full reimbursement of innovative treatments, including gene therapies, by the third-party social insurance payer, i.e., the National Organization for Health Services Provision (EOPYY), there persists a lack of definition of an optimal patient pathway that enhances patient experience with the system and, ultimately, improves care outcomes.

This survey shares the journeys of parents of children with SMA through care in Greece. It details the challenges faced in each stage of this journey, by detailing what parents had to do, what they had to pay, and how they felt. It also used validated scales (PedsQol, Barthel index, and the Zarit scale) to correlate areas of quality of life of the child and ability to perform activities with the level of strain placed on parents, as well as record defined values in each index that would assist benchmarking across diseases, primarily rare, within Greece and beyond. Finally, the study provides a platform to support the definition of actionable priorities for health policy advocacy at the healthcare system level on the basis of actual evidence from parents of children with SMA.

## 2 Materials and methods

The study recruited adult parents of children with an SMA diagnosis who, at the time of study, were members of MDA Hellas. Dissemination was the sole responsibility and decision of MDA Hellas. Participants were sent an invitation directly by MDA to participate in the survey by completing an online, novel questionnaire through the Jotform platform. All responses were collected by MDA Hellas. Only one of the two parents of each child could participate in the survey. This approach was deemed appropriate to ensure data could be gathered concurrently by all participants in a structured manner.

### 2.1 Survey tool

The questionnaire was developed and customized to the survey audience, i.e., to parents and/or unpaid caregivers of pediatric patients. The questionnaire was conditioned by the specifics of the Greek healthcare system, which is plagued by a lack of integration of healthcare services, suboptimal care networks, and informal, out-of-pocket payments, over and above the formal reimbursement of care services by EOPYY, which are reported as persistently higher than the EU average (13). Prior to finalizing the questionnaire, we held two unstructured, lengthy interviews with two parents of children with SMA, members of MDA, to ensure the relevance of all questionnaire sections to their major challenges and concerns. Section 1 of the questionnaire recorded basic sociodemographic characteristics and disease history. Section 2 mapped the full patient journey across a continuum, per disease phase, i.e., (a) before and during diagnosis and (b) from treatment start to disease management to date. In addition, the participants were requested to assess their child's status regarding functional autonomy, using the validated Barthel index scale (14). Moreover, they assessed the overall quality of life of the child using the Pediatric Quality of life (PedsQol) parent/carer proxy reports (15, 16) and their own burden, using the Zarit scale (17). Finally, the questionnaire recorded disease-related personal or family financial burden. The latter included financial burden due to travel from the place of residence to access services, the cost of disease-related healthcare services, and aids that the parents had to pay out of their pocket, if not reimbursed, either in part or in full, by EOPYY. For all expenditures, the mean value was computed by summing the corresponding cost across parents who had reported this cost and dividing it by the number of parents who reported it. Cronbach's alpha reliability index was 0.83 for the Zarit scale and 0.90 for the Barthel index scale. For PedsQol, reliability indexes ranged from 0.71 to 0.85, indicating acceptable reliability of all scales.

### 2.2 Data management processes

The Health Policy Institute prepared the online survey questionnaire, which was reviewed and approved by MDA Hellas. The questionnaire was fully anonymized, and responses could not be traced back to participants by those performing the analysis. Great attention was paid to promoting the use of ranges to responses to all questions for which this was deemed appropriate for this type of survey, to ensure any risk of re-identifying is minimized. The Health Policy Institute, which was scientifically responsible for the analysis of responses, never collected, acquired access to, stored, or processed non-anonymized respondent data that were not provided by MDA Hellas.

### 2.3 Pilot survey

Questionnaires were sent by MDA Hellas to four parents of pediatric patients to pilot completion and test content validity and completeness. As there were no suggestions for further improvements of the questionnaire, pilot survey responses were accounted for in the total survey sample.

### 2.4 Statistical analysis

To describe the data, quantitative variables were expressed as mean values (standard deviation) and as median (interquartile range), while categorical variables were expressed as absolute and relative frequencies. To investigate the correlation between areas of quality of life of the child and functional capacity with parental burden, we used Pearson’s correlation coefficient (*r*). In addition, to investigate the differences in children's QoL, functional capacity, and parental burden among types of SMA, we used analysis of variance (ANOVA). To investigate the association of parental needs with their educational level and their annual income, we used Pearson's chi-square tests or Fisher's exact tests. All reported *p*-values are two-tailed. Statistical significance was set at *p* < 0.05, and analyses were conducted using Statistical Package for the Social Sciences (SPSS) software (version 26.0).

## 3 Results

### 3.1 Sample sociodemographic characteristics, disease background, and treatment patterns

Forty-four parents, each representing one family, participated in the survey, out of a total of 56 parents who were members of MDA Hellas at the time of the study. The mean age of parents was 43.2 years (SD = 7.1 years). Their characteristics, along with those of their children, are presented in Table 1. Most participants were women (70.5%), university alumni (45.5%), and married (95.5%), had an annual family income of 10,000–30,000 euros (59.1%), lived with the child (97.7%), and had to accompany the child to all medical/disease management appointments (97.7%). The majority (88.5%) reported taking time off work to attend to the child’s needs. Specifically, 34.8% had to take time off work once every 3 months, while 26.1% did so once a month. The children had a mean age of 8.5 years (SD = 4.6 years), with a mean time from diagnosis of 7.1 years (SD = 4.2 years). Half of the children (50%) had been diagnosed with type II disease, 90.9% were currently under treatment, and 54.5% (*N* = 24) received their treatment once every 4 months. Furthermore, 27.3% of children had experienced a treatment-related adverse event, and 25.0% of them were hospitalized for this event.

**Table 1:** Parents' and children's characteristics.

*n* = 44	*n*	%
Parents' characteristics		
Gender		
Men	13	29.5
Women	31	70.5
Educational level		
Middle school	2	4.5
High school	12	27.3
University	20	45.5
MSc/PhD	10	22.7
Employment status		
State employee	10	22.7
Private employee (full-time)	10	22.7
Private employee (part-time)	2	4.5
Freelance	3	6.8
Household	6	13.6
Unemployed	12	27.3
Other	1	2.3
Family status		
Married	42	95.5
Divorced	2	4.5
Annual family income (euros)		
<10,000	13	29.5
10,000–30,000	26	59.1
30,000–60,000	5	11.4
Private insurance^c^	14	31.8
What is the distance from the hospital (e.g., pediatric neurology clinic) that attends the patient? (km)		
<10	9	20.5
10–50	19	43.2
50–100	6	13.6
>100	10	22.7
Has your family situation changed since your child was diagnosed with spinal muscular atrophy (i.e., separated, divorced, and married)?^a^	10	22.7
Has there been a change in your employment since your child was diagnosed with spinal muscular atrophy?^b^		
I have stopped working	1	3.8
I have reduced my working hours	6	23.1
No change	17	65.4
I have increased my working hours	2	7.7
Do you live with the child?^a^	43	97.7
Do you accompany the child to all medical/disease management appointments?^a^	43	97.7
Do you take time off work to be present when the child needs anything?^a^,^b^	23	88.5
On average, how long is the absence from work each time?^b^		
One day	12	52.2
Some days	10	43.5
One week	1	4.3
Missing	3	
Children's characteristics	Age, mean (SD)	
Years from diagnosis, mean (SD)	7.1 (4.2)	8.5 (4.6)
Type		
Ι	15	34.1
ΙΙ	22	50.0
ΙΙΙ	7	15.9
Currently under treatment^a^	40	90.9
Frequency of treatment		
Daily	12	27.3
Once every 4 months	24	54.5
Other	8	18.2
To date, what types of treatment has the child received?^c^		
Gene therapy	16	36.4
Disease modifiers	20	45.5
Management of symptoms only	12	27.3
Other	9	20.5
Did the child experience any side effects from the treatment?	12	27.3
Management of side effects required hospitalization?^d^	3	25.0

SD, standard deviation.

^a^ Possible answers were yes/no. Numbers provided refer to “yes.”

^b^ Refers to parents who work (*n* = 26).

^c^ Participants could select more than one answer.

^d^ Refers to cases with reported treatment side effects (*n* = 12).

### 3.2 Journey phase I: diagnosis

SMA was diagnosed through newborn screening in only 22.7% of the total sample (*N* = 10), while no prenatal screening cases were reported by participants. In the remaining cases, the first symptom was primarily identified by the parent (73.5%). Post symptom identification, 73.5% of respondents contacted a pediatric neurologist, and 70.6% underwent diagnostic tests, of which 95.8% confirmed the child's diagnosis. In the majority of cases (52.9%), the diagnosis was confirmed within 3 months of first seeking care.

More than one in three (38.6%, *N* = 17) were required to travel from their place of residence to receive a diagnosis, and all (*N* = 17) covered this cost out of their pocket. Among those required to travel to receive a diagnosis, 70.6% had to travel for more than 100 km. Of the 26 parents in the sample who were employed at the time of the survey, 19 (73.1%) had to take leave of absence from their work to receive a diagnosis, and 42.1% of these had to take more than 3 days off work.

In addition, more than half (65.9%) had to pay out-of-pocket for diagnosis-related services (Table 2), primarily diagnostic tests (82.8%) and medical visits (69.0%). The majority of children (86.4%) started their treatment more than 1 month after confirming the diagnosis. The most frequent reason (76.9%) for this delay was getting treatment approval by EOPYY.

**Table 2:** Diagnosis-related services paid out-of-pocket and barriers in receiving treatment.

Variables	*n*	%
Did you have to pay out-of-pocket for any health services related to the diagnosis?^a^	29	65.9
If yes, for which one?^b^^,^^c^	15	34.1
Medical visit	20	69.0
Medical visit (co-payment only)	3	10.3
Diagnostic tests	24	82.8
Diagnostic tests (co-payment only)	6	20.7
Pharmaceutical treatments	3	10.3
Pharmaceutical treatments (co-payment only)	0	0.0
Other	1	3.4
In how many days after the diagnosis did the child start treatment?		
1 week	1	2.3
15 days	2	4.5
1 month	2	4.5
>1 month	38	86.4
Other	1	2.3
Did you experience problems/delays with starting the (drug) treatment?^a^	26	59.1
If yes, define^c^,^d^		
Treatment approval (SEP/EOPYY)	20	76.9
Availability of treatment (e.g., delayed importation from abroad)	9	34.6
Availability of hospital/clinic (appointment) for the administration of the treatment	9	34.6
Other	4	15.4

EOPYY, national organization for health services provision; SEP, system for electronic preauthorization.

^a^ Possible answers were yes/no. Numbers provided refer to “yes.”

^b^ Refers to parents who paid out-of-pocket for services related to the diagnosis (*n* = 29).

^c^ Participants could select more than one answer.

^d^ Refers to cases that experienced a delay in treatment start (*n* = 26).

**Figure 1:**
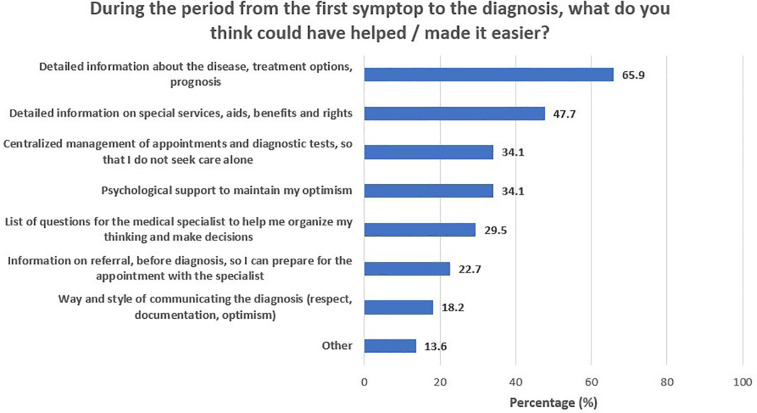
During the period from the first symptom to the diagnosis, what do you think could have helped/made it easier?

**Figure 2:**
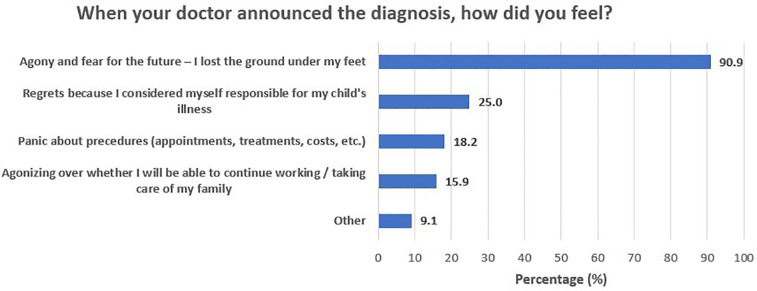
When your doctor announced the diagnosis, how did you feel?

During the period from first symptom recognition to treatment start, parents reported a heavy emotional burden and underlined a need for accurate, comprehensive, and timely information provision. Figure 1 depicts participants’ self-reported needs and requests for support during this phase of the journey. A total of 65.9% of participants reported that detailed information on the disease, treatment options, and prognosis could have helped them manage the fear and anxiety that came with the diagnosis. Another 47.7% of parents stressed the importance of having easy access to detailed information on special services and assistive equipment/aids, social insurance benefits, and patient rights, whereas one in three (34.1%) would welcome centralized organization of the various diagnosis-related appointments and tests. Finally, an additional 34.1% stressed the need for psychological support for parents and carers, to assist them in maintaining their optimism. The need for information about the disease, treatment options, and prognosis was similar across parental education (64.3% for middle/high school graduates and 66.7% for university alumni/MSc/PhD holders; *p* > 0.999) and income levels (53.8% for those with less than 10,000 euros annual income and 71.0% for those with more than 10,000 euros annual income; *p* = 0.313) (Table 3). Furthermore, need for information on special services, aids, benefits, and rights was significantly greater in university alumni/MSc/PhD holders (21.4% for middle/high school graduates and 60.0% for university alumni/MSc/PhD holders; *p* = 0.017), yet similar across income levels (38.5% for those with less than 10,000 euros annual income and 51.6% for those with more than 10,000 euros annual income; *p* = 0.426). In addition, the need for referral information, before diagnosis, so that they can be prepared for the appointment with the specialist was similar across parental education (21.4% for middle/high school graduates and 23.3% for university alumni/MSc/PhD holders; *p* > 0.999) and income levels (23.1% for those with less than 10,000 euros annual income and 22.6% for those with more than 10,000 euros annual income; *p* > 0.999). Figure 2 presents the feelings reported by participants upon receiving the diagnosis. The majority of participants (90.9%) felt agony and fear for the future, and one in four (25%) felt guilt, as they considered themselves responsible for their child's illness.

**Table 3:** Association of parental needs with their educational level and their annual income.

Variables	Need for information about the disease, treatment options, and prognosis		Need for information on special services, aids, benefits, and rights		Need for information on referral, before diagnosis, so that parents can be prepared for the appointment with the specialist	
	*n* (%)	*p*	*n* (%)	*p*	*n* (%)	*p*
Educational level						
Middle/high school graduates	9 (64.3)	>0.999^b^	3 (21.4)	0.017^a^	3 (21.4)	>0.999^b^
University alumni/ MSc/PhD holders	20 (66.7)		18 (60)		7 (23.3)	
Family's annual income (euros)						
<10,000	7 (53.8)	0.313^b^	5 (38.5)	0.426^b^	3 (23.1)	>0.999^b^
≥10,000	22 (71)		16 (51.6)		7 (22.6)	

^a^ Pearson's chi-square test.

^b^ Fisher's exact test.

### 3.3 Journey phase 2: linkage to care and treatment

Physiotherapy/occupational therapy was used by 86.4% of the children and orthopedic monitoring/tests [e.g., dual-energy x-ray absorptiometry (DEXA)] by 43.2% (Table 4). Speech therapy and orthopedic monitoring/tests were the two types of expenses most frequently reported as reimbursed by EOPYY, in 50% and 47.4% of the sample, respectively. In those cases, parents had to shoulder a monthly expense of on average 319.5 euros (SD = 154.1 euros) for physiotherapy/occupational therapy and 185.7 euros (SD = 146.8 euros) for speech therapy. Two out of three children (66.7%) used orthotic aids and 79.4% splints, none of which were fully reimbursed by EOPYY (Table 5). Moreover, 75% of the children used a wheelchair, which was fully reimbursed in only 9.5% of the cases. In total, the overall cost that parents had to shoulder, as it was not reimbursed by EOPYY, ranged from 80 to 166,238€, with a mean of 8,371.11€ (SD = 28,329.09€) and a median of 1,450€ (IQR: 350–4,680€).

**Table 4:** Parents' out-of-pocket expenses.

Type of expense^b^	Children’s use^a^		Expense reimbursed by EOPYY^a^		Monthly out-of-pocket amount spent (€), if not reimbursed by EOPYY	
	*n*	%	*n*	%	Mean (SD)	Median (IQR)
Food supplements/vitamins	19	43.2	6	31.6	71.4 (66.8)	30 (20–100)
Physiotherapy/occupational therapy	38	86.4	16	42.1	319.5 (154.1)	285 (200–450)
Speech therapy	14	31.8	7	50.0	185.7 (146.8)	120 (100–240)
Nutritional monitoring/assessment/intervention	10	22.7	2	20.0	86.3 (64.1)	60 (35–135)
Orthopedic monitoring/tests (e.g., DEXA)	19	43.2	9	47.4	53 (27.5)	50 (30–60)
Dental/orthodontic follow-up/intervention (e.g., due to jaw problems)	12	27.3	2	16.7	50 (44.7)	45 (20–50)
Psychological support (for me or my partner)	6	13.6	1	16.7	84 (70.2)	50 (40–100)
Physiotherapy (for me or my partner)	14	31.8	6	42.9	178.8 (183.3)	150 (45–200)
Other	2	4.5	0	0.0	55 (63.6)	55 (10–100)

DEXA, dual-energy x-ray absorptiometry; EOPYY, national organization for health services provision; IQR, interquartile range; SD, standard deviation.

^a^ Possible answers were yes/no. Numbers provided refer to “yes.”

^b^ Participants could select more than one answer.

**Table 5:** Aids used by children and their reimbursement.

Type of aid^d^	Have it at home^a^	If yes, was the cost of buying it reimbursed by EOPYY?^b^			Out-of-pocket amount spent (€)^c^
		No	Partly	In full	
	*n* (%)	*n* (%)	*n* (%)	*n* (%)	Mean (SD)
Simple continuous positive airway pressure (CPAP) device	5 (20)	0 (0)	1 (33.3)	2 (66.7)	4,500 (—)
Missing	19		2		
Simple biphasic positive airway pressure (BiPAP S/T) device	15 (48.4)	1 (9.1)	6 (54.5)	4 (36.4)	1,736.7 (2,586)
Missing	13		4		1
Volume/pressure ventilator	11 (37.9)	0 (0)	5 (71.4)	2 (28.6)	125 (35.4)
Missing	15		4		3
Orthotic means	20 (66.7)	2 (11.1)	13 (72.2)	3 (16.7)	1,261.8 (468.3)
Missing	14		2		4
Knee guards	12 (42.9)	5 (45.5)	5 (45.5)	1 (9.1)	564.3 (857.7)
Missing	16		1		3
Shoulder guards	5 (20)	2 (40)	3 (60)	0 (0)	1,633.3 (2,064.8)
Missing	19				2
Splints	27 (79.4)	3 (13)	20 (87)	0 (0)	800 (1,027.6)
Missing	10		4		5
Wheelchair	27 (75)	3 (14.3)	16 (76.2)	2 (9.5)	2,516.7 (1,977.8)
Missing	8		6		4
Special van/vehicle that allows the transportation of a wheelchair	4 (14.8)	4 (100)	0 (0)	0 (0)	95,000 (91,923.9)
Missing	17				2

BiPAP, biphasic positive airway pressure; CPAP, continuous positive airway pressure; EOPYY, national organization for health services provision; SD, standard deviation; S/T, spontaneous/timed.

^a^ Possible answers were yes/no. Numbers provided refer to “yes.”

^b^ Answered only by participants who had aid at home.

^c^ Answered only by participants who stated that the cost was not reimbursed by EOPYY either in full or in part.

^d^ Participants could select more than one answer.

### 3.4 Correlation of areas of quality of life of the child and functional capacity with parental burden

PedsQol scales' descriptives and their correlation with the Barthel index and Zarit scale are presented in Table 6. Children's quality of life was not significantly correlated with their functional capacity (as measured by the Barthel index). On the contrary, a higher score in the Zarit scale, i.e., greater burden, was significantly associated with lower physical functioning, lower social functioning, and lower QoL overall. Mean functional capacity was 37.7 (SD = 26.7), and mean burden was 14.1 (SD = 7.5). Higher functional capacity in children was significantly associated with lower parental burden (*r* = −0.52; *p* < 0.001).

**Table 6:** PedsQol scales' descriptives and their correlation with the Barthel index and Zarit scale.

Variables	Mean (SD)	Barthel index		Zarit scale	
		*r*	*p*	*r*	*p*
Physical functioning	68.9 (28)	0.14	0.379	−0.31	0.041
Emotional functioning	69.9 (17.7)	0.11	0.482	−0.29	0.056
Social functioning	58.1 (22.6)	0.04	0.778	−0.37	0.013
School functioning	71 (24.1)	−0.03	0.848	−0.04	0.815
Total PedsQol score	67 (15.8)	0.09	0.540	−0.36	0.015
Barthel index	37.7 (26.7)			−0.52	<0.001

PedsQol, pediatric quality of life; SD, standard deviation.

### 3.5 Differences in children’s QoL, functional capacity, and parental burden among types of SMA

To examine if children's QoL, functional capacity, and parental burden differed between SMA types, we compared all scale scores across the three SMA types (Table 7). The mean functional capacity score was 15.3 (SD = 14.3) in participants with SMA type I, 38.6 (SD = 14.4) in SMA type II, and 82.9 (SD = 16.0) in SMA type III. Functional capacity was significantly improved in type III compared with types I and II, and significantly greater in type II compared with type I (*p* < 0.001 for all comparisons). The mean score on the burden scale was 19.9 (SD = 7.1) for participants with SMA type I, 12.2 (SD = 5.1) for participants with SMA type II, and 7.4 (SD = 6.9) for participants with SMA type III. Parental burden was significantly lower in children with SMA type III as compared with types I (*p* < 0.001) and II (*p* = 0.002). QoL was not significantly different among the different SMA types (*p* > 0.05).

**Table 7:** PedsQol scales, Barthel index, and Zarit scale according to SMA type.

Variables	SMA type			*p* ANOVA	*p* ^a^		
	Ι	ΙΙ	ΙΙΙ				
	Mean (SD)	Mean (SD)	Mean (SD)		I vs. II	I vs. III	II vs. III
Barthel index	15.3 (14.3)	38.6 (14.4)	82.9 (16.0)	<0.001	<0.001	<0.001	<0.001
Zarit scale	19.9 (7.1)	12.2 (5.1)	7.4 (6.9)	<0.001	0.002	<0.001	ns
Physical functioning	59.2 (35.3)	72.9 (25)	77.2 (12.7)	0.243	ns	ns	ns
Emotional functioning	70.3 (16.7)	70.7 (18)	66.4 (21.2)	0.858	ns	ns	ns
Social functioning	54.3 (27.8)	61.1 (18.6)	56.4 (23.8)	0.663	ns	ns	ns
School functioning	71.4 (28.8)	70.5 (21.9)	71.9 (23.4)	0.988	ns	ns	ns
Total PedsQol score	63.8 (19.1)	68.8 (13.3)	68 (16.9)	0.645	ns	ns	ns

^a^ *p*-value from pairwise comparisons (after Bonferroni correction).

## 4 Discussion

Our results confirm that, in the majority of cases and given the lack of organized prenatal or newborn screening, parents are the first to recognize any symptoms and seek care. Over one-third of parents have to travel from their place of residence to receive a diagnosis for their child, and more than half have to pay out-of-pocket for diagnosis-related services. In addition, parents shoulder treatment services and assistive aids' costs in excess of 300 euros a month. The majority report feelings of agony, fear, and guilt upon receiving the diagnosis. Caregivers of children with severe SMA types report a higher burden.

Our findings confirm results from a previous study, in which among types I, II, and III patients, 63%, 75%, and 85% of cases were first recognized by parents, respectively (18). In the same study, the mean time between symptom onset and genetic diagnosis was 1.94 months (SD ± 1.84; range: 0–10.3 months) in type I patients, 5.28 months (SD ± 4.68; range: 0–35 months) in type II, and 16.8 months (SD ± 18.72; range: 0–102 months) in type III (18), which is longer than the average of 3 months reported in our study. Lack of specialized knowledge to recognize symptoms or even assert a behavior as abnormal by parents may be related to a delayed diagnosis (19), which inevitably impacts the prospects of successful early intervention. A recent systematic review (20) revealed that many parents described an early feeling that something was wrong with their child—nonetheless, most of them received the diagnosis with a delay.

The majority of our sample reported feelings of shock, loss, and guilt upon receiving the formal diagnosis. Such sentiments are reported as related to the confrontation with the possible premature death of the child and lost expectations for a normal life (20). This “anticipatory loss’s” main themes include (1) enduring the helplessness and pressure of care, (2) suffering due to the child's rare and unknown condition, (3) loss of hope and reinforcement of the parent–child attachment, and (4) avoiding the pressure of death and enriching the child's life (21).

In this light, parents' most pressing need, mentioned consistently in the literature and confirmed by our survey, is information (22–27), particularly about treatment options. The need for information about the child's options, prospects, and the institutions that can benefit the child repeatedly comes to the fore and, in the literature, appears to be unrelated to the educational level of the mother or father and related to family income, with the need for information relatively decreasing as income increases (22). Likewise, our survey highlights that parental need for information appears unrelated to educational level, except for specialized information on special services, aids, benefits, and rights, which was significantly greater in university alumni/MSc/PhD holders. This may be explained by the fact that parents with a higher education may be better positioned to fully understand and advocate for their child's rights. On the contrary, our survey revealed that parental need for information is wholly unrelated to family income. This may be explained by the fact that the disease is rare and all parents, irrespective of income, are faced first and foremost with the same need for information. Another recent review (20) revealed that parents express a consistent need for coordinated and integrated care, including not only medical treatment but also psychological and practical care support, from very early on, from the point of diagnosis. These findings are fully reflected in our study. Parents often report feeling left alone after the diagnosis, struggling to find the right solutions for their child, because of complex and time-consuming navigation through the healthcare and support system (20). The lack of clearly defined and integrated support pathways in the current structure and operation of the Greek National Health System (NHS) further confounds this situation and is confirmed by participants in this study as of critical importance both at the stage of diagnosis and during the ongoing management of pediatric SMA.

Out-of-pocket expenditure is also a major worry for parents of pediatric SMA patients. Shouldering a cost that may exceed 300 euros a month for a series of additional health services or assistive aids and equipment places a substantial burden not only on the financial but also on the psychological capacity of parents. With challenges related to maintaining employment despite the need for constant leave of absence and the increasing requirements for support services as the child grows, the fear of being unable to make ends meet is prevalent in most survey respondents. Particularly for some expenses, such as wheelchairs and vans, for which the respective out-of-pocket expenditure could be considered as catastrophic (28). Being unable to shoulder such costs may, in addition, impact actual disease outcomes. Our study confirmed that children, whose parents had shouldered out-of-pocket expenses as early as the diagnosis phase, had significantly improved school functioning scores. In a study of Thai children with SMA (29), whose health-related quality of life (HRQoL) was compared with those of healthy Thai children, household income less than 18,500 Thai baht/month was statistically significantly associated with lower HRQoL, as costs of assistive devices that allow these patients to live and function more independently were not covered by Thailand's universal healthcare coverage insurance scheme. Although not confirmed by our data due to the sample size, this aspect warrants careful consideration in settings where access to assistive devices is limited or problematic, as reported in this survey for Greece.

Indirect costs, associated with income loss, are also highlighted in our study, across the disease continuum, for those parents who can maintain their employment post-diagnosis. This is in line with a recent review of the economic impact of SMA across Europe (30), which highlighted that direct non-healthcare costs ranged between a whopping 79%–86% of total SMA costs, with informal care costs being the main component. This confirms older results from Spain (31) that attributed 67.8% of total SMA costs to direct non-healthcare costs. Of these, informal caregiving constituted the major cost component.

Among our sample, the average PedsQol score was 67 (SD = 15.8), which is higher than scores recently reported in Thailand (54.3 ± 14.8) (29) and Chile (51.92 ± 17) (32). Zarit scores per SMA type were higher for type I SMA vs. types II and III, revealing a higher burden for caregivers in the graver SMA types. These results are substantially different from those recently reported in Aranda-Reneo et al. (33) [25.1 (23.2) for type I, 34.8 (13.3) for type II, and 29.6 (17.1) for type III]. These differences, especially when considering Zarit scores by SMA type, may be attributed to the low participant numbers in such surveys, which may lead to biases when extrapolating to the entire SMA population.

## 5 Limitations

All data in this study are self-reported. Some refer to past events and may, thus, be affected by recall bias, as some of the costs (e.g., aids and supportive equipment) incurred out of pocket or self-reported quality of life may be affected by participants' incomplete memories. This recall bias may impact the true relationship between variables.

The survey was performed in Greece through MDA Hellas to ensure patient concerns and perspectives are input into formal health policy advocacy. As such, results may be conditioned by personal patient experiences with the Greek healthcare system’s specifics regarding funding and access to care. Nonetheless, the survey is replicable in other countries, through a patient association or an advocacy organization, and as such can provide insights into similarities and differences in perceptions and challenges faced by parents of SMA children in different healthcare systems.

Participants, all parents of children diagnosed with SMA, were members of MDA Hellas in Greece. For this study, only one of the two parents was included to accurately reflect the number of children represented in the study. Our sample size of 44 parents may, therefore, be considered representative for a study among parents of underage children with such a rare disease as SMA in Greece.

## 6 Conclusions

Given the nature of SMA as a rare genetic disorder, pediatric patients and their caregivers are faced with additional challenges as they navigate their journey, related primarily to the rarity and severity of the condition. This paper details their concerns and needs at each phase of their patient journey to ensure these can be measurably addressed through targeted health policy advocacy. Such advocacy should be translated into a national action plan, which adequately addresses these specific patient and caregivers' needs, as highlighted in this study. In addition, future studies are required to follow patients over time to understand long-term effects on caregivers and the impact of different SMA treatment modalities or care organization and delivery options.

## Data Availability

The raw data supporting the conclusions of this article will be made available by the corresponding author, upon request.
